# Who Provides Home-based Primary Care to People with Dementia?

**DOI:** 10.1093/geroni/igaf122.2535

**Published:** 2025-12-31

**Authors:** Monica O’Reilly-Jacob, Lusine Poghosyan, Madison Horton, Carolyn Clevenger, Soo Borson, Jianfang Liu

**Affiliations:** Columbia University School of Nursing, Andover, Massachusetts, United States; Columbia University, New York, New York, United States; Columbia University, New York, New York, United States; Emory University, Atlanta, Georgia, United States; University of Southern California, Los Angeles, California, United States; Columbia University Irving Medical Center, New York City, New York, United States

## Abstract

Home-based primary care (HBPC) delivery for people with dementia (PWD) is growing. Nurse practitioners (NPs) and physicians (MDs) deliver HBPC, yet how to optimize workforce resources in HBPC models is unknown. We used national 2019 Medicare claims to examine the characteristics of PWD receiving HBPC from NPs, MDs, or both from NPs and MDs. We identified PWD over age 65 who received > =4 HBPC visits extracted age, dual-eligibility for Medicare and Medicaid, race/ethnicity and 14 clinically meaningful chronic conditions. We assigned each beneficiary to a HBPC model (i.e., NP-only, MD-only, NP-MD) depending on the type of clinician providing the totality of HBPC visits. We used analysis of variance or Chi-square analyses to assess group differences. In 2019, 62,094 Medicare beneficiaries with dementia received at least four HBPC visits. Overall, almost 40% received HBPC exclusively from NPs, 33% from MDs, and 26% from NPs and MDs. Those seen by both NPs and MDs had significantly higher rates of almost all chronic conditions (p < .001) and were significantly more racially and ethnically diverse (Non-Hispanic White: 58.3%) than those that saw NPs or MDs exclusively (Non-Hispanic White: NP-only: 71.1%; MD-only: 65.2%, p < .001). The majority of HBPC for PWD is provided by NPs. HBPC provided by both NPs and MDs is more likely delivered to those with more chronic conditions and of racial and ethnic minorities. Leveraging the expertise of both NPs and MDs in HBPC models may be necessary for homebound PWD of greater clinical complexity.

